# Supplementary material for: Complexome profiling of the Chlamydomonas psb28 mutant reveals TEF5 as an early PSII assembly factor
Source: Plant Cell. 2025 Mar 17;37(6):koaf055. doi: 10.1093/plcell/koaf055 (PMC12164590; doi:10.1093/plcell/koaf055)
Supplement: koaf055_Supplementary_Data [file koaf055_supplementary_data.zip › psb28_interactive.zip!/plots/intensity/qualityPlots/peptides/NUO13_Cre11.g467767.t1.1.html]

### Cre11.g467767.t1.1

Trivial name: NUO13  
SeqCoverage [median]: 46.80  
Mass: 18.01 kDa  
  
 

| Sequence | MissedCleavage | Mass [Da] | wtIntensity | muIntensity | Score |
| --- | --- | --- | --- | --- | --- |
| KYESWTPANK | 1 | 1222.60 | 0.00 | 2898.57 | 60.43 |
| LLEASGK | 0 | 716.41 | 0.00 | 2582.12 | 92.47 |
| LLEASGKK | 1 | 844.50 | 5135.73 | 8100.94 | 116.73 |
| LVGTDYNGNR | 0 | 1107.53 | 6226.80 | 3844.70 | 97.80 |
| MLVDGNLAHNVLVR | 0 | 1549.84 | 25639.95 | 35981.83 | 99.50 |
| QPTYQIEASMTK | 0 | 1395.67 | 7551.59 | 3298.48 | 144.17 |
| SLSGYFSR | 0 | 915.45 | 15937.58 | 4943.98 | 119.41 |
| YESWTPANK | 0 | 1094.50 | 495.45 | 461.71 | 97.80 |
| YYENEEAAYGR | 0 | 1363.57 | 10612.16 | 9986.59 | 194.06 |
|  |  |  | 71599.3 | 72098.9 |  |
